# Spinal cord injury is associated with reduced cognitive capacity to self-regulate, organise and adapt to new situations

**DOI:** 10.1038/s41393-026-01264-4

**Published:** 2026-09-02

**Authors:** Daniel Myles, Danielle Sandalic, Michael Gascoigne, Ilaria Pozzato, Mohit Arora, Candice McBain, Yvonne Tran, James W. Middleton, Ashley Craig

**Affiliations:** 1 John Walsh Centre for Rehabilitation Research, Kolling Institute, Northern Sydney Local Health District, St. Leonards, NSW 2065 Australia; 2 Faculty of Medicine and Health, The University of Sydney, St Leonards, NSW 2065 Australia; 3 School of Psychology & Translational Health Research Institute, Western Sydney University, Sydney, Australia

**Keywords:** Spinal cord diseases, Human behaviour

## Abstract

**Study design:**

Cross-sectional observational cohort study

**Objectives:**

To investigate differences in executive function (EF) in adults with spinal cord injury (SCI) compared with able-bodied (AB) controls across tasks imposing varying levels of executive demand, and to examine implications for targeted cognitive screening.

**Setting:**

Two SCI units in New South Wales (SCI), Australia, and the community of New South Wales, Australia (able-bodied).

**Methods:**

Executive function was assessed in 40 adults with acute SCI and 32 AB controls using the Neuropsychiatry Unit Cognitive Assessment Tool (NUCOG). Two multivariate analyses of covariance (MANCOVA) were conducted to compare group performance across tasks requiring higher-order EF processes and tasks characterised by minimal EF demand, controlling for age, sex, and years of education.

**Results:**

A significant multivariate group difference was observed across EF tasks, with individuals with SCI demonstrating significantly lower performance on categorical fluency, abstraction, and Digit Span Reverse following correction for multiple comparisons. No significant group differences were observed across tasks characterised by minimal EF demand.

**Conclusion:**

Adults with SCI exhibit selective impairments in higher-order executive functioning that emerge under increased cognitive demand, while performance on low-demand tasks remains relatively preserved. These findings highlight the importance of assessing EF components at the task level and support the need for targeted screening approaches sensitive to higher-order executive deficits to optimise rehabilitation outcomes.

## Introduction

Spinal cord injury (SCI) has been found to be associated with higher rates of mild cognitive impairment (MCI), which is estimated to affect around 30% of those with acute SCI [1]. Research suggests adults with SCI have almost 18 times the odds of having MCI compared to adults without SCI [2]. MCI is a syndrome that can be described by decline in cognitive function, such as attention, learning and memory, executive function, processing speed, and semantic language, that exceeds the expected level of decline for a person’s age and level of education [3]. A meta-analysis provided an improved understanding of MCI after SCI by synthesising research across the five cognitive functioning domains that subtype neurocognitive disorders according to the Diagnostic and Statistical Manual of Mental Disorders Fifth Edition [4]. The meta-analysis established that of the five cognitive domains, executive function (EF) and attention were the most seriously affected [5]. These domains being most affected is not surprising given the interplay between attention and one’s ability to perform EF based tasks, whereby difficulties in one will likely create challenges in the other [4–6].

EF’s are described as a family of top-down mental processes essential for tasks requiring focused attention, self-regulation, planning, organization, and adaptability to changing situations [7, 8]. Three core EFs are inhibition (inhibitory control and self-control), working memory, and cognitive flexibility (set shifting and mental flexibility) [7, 8]. The meta-analysis findings are consistent with aging literature that shows age-appropriate cognitive decline to involve decreased neural activity in localised brain regions such as the prefrontal cortex [9, 10], a key brain region involved in executive control of important daily functions [9, 11].

Neurophysiological measures of brain activity provide insights into broader neurological differences associated with SCI. Electroencephalogram (EEG) studies of brain wave activity comparing adults with SCI and able-bodied (AB) participants have shown lower magnitude and peak amplitude in the α wave frequency (8–13 Hz), with the largest differences found in the central, parietal, and occipital sites for those with SCI [12, 13]. These EEG studies showed that adults with SCI experience a shift in EEG towards slower brain frequencies (theta and low alpha bands) typical of accelerated ageing [12]. Lazzaro et al. support this finding by showing that adults with SCI have significantly reduced P300 amplitudes when compared with AB controls [14]. The amplitude of the P300 signal is believed to be proportional to the amount of attentional resources devoted to a task and the degree of information processing required, suggesting reduced EF capacity in the areas of inhibitory control, working memory, and cognitive flexibility [14].

Furthermore, increased slow-wave activity and reductions in faster frequency bands have been reported in adult neurological and neurodevelopmental conditions associated with attentional and EF inefficiencies [15–17]. Such findings suggest that a relative shift toward slower cortical rhythms may reflect reduced neural efficiency and disrupted large-scale network regulation underlying executive control [15, 16]. Consistent with this interpretation, faster alpha-band activity has been associated with more robust executive functions, including planning, organisation, and cognitive flexibility, whereas a predominance of slower frequencies has been linked to diminished cognitive control [16, 17]. These findings highlight the need to understand how EF deficits affect performance on cognitive tasks essential for successful SCI rehabilitation.

This study aims to build on existing research by examining EF performance in adults with SCI by comparing them with AB control on tasks of varying EF demands. The objectives of this study are as follows:

(i) Investigate whether adults with SCI and AB participants differ in performance on tasks involving high EF demands.
(ii) Evaluate performance of adults with SCI and AB participants on tasks requiring minimal EF demands.

By addressing these objectives, this study seeks to provide a clearer understanding of executive functioning performance in adults with SCI across tasks imposing varying executive demands.

## Methods

This cross-sectional observational cohort study was conducted in New South Wales, Australia. Ethical approval for recruiting adults with SCI was granted by the Northern Sydney Local Health District Human Research Ethics Committee (2019/ETH00592), and approval for recruiting AB participants was obtained from the Navitas Professional Institute Human Research Ethics Committee (NPI-HREC; 754010921). The study protocol involving adults with SCI has been published elsewhere [18] and was prospectively registered with the Australian and New Zealand Clinical Trials Registry (ACTRN12619001702101) on December 3, 2019. All participants provided electronically signed informed consent prior to enrolment.

### Participants

This study was comprised of two groups: adults with SCI (*SCI group*, a sub-cohort of the larger study) and an AB cohort with similar demographics (*AB group*).

The SCI group consisted of 40 acute patients with SCI admitted to two SCI units in the NSW between 2019 and 2022. Inclusion criteria consisted of 1) the presence of an acute SCI from traumatic or non-traumatic aetiology, (2) SCI of any extent (complete or incomplete) and level (paraplegia or tetraplegia), and 3) aged 17-80 years and of either sex. Exclusion criteria included: 1) insufficient proficiency in the English language to complete self-report measures and 2) the presence of a severe mental health disorder (e.g., schizophrenia or bipolar disorder) and/or a physical illness, such as severe traumatic brain injury or dementia. The SCI sample included in the present study represents a sub-cohort of a larger dataset previously reported by Arora et al. [1]. The reduced sample size reflects the inclusion of participants who completed the full cognitive assessment battery required for the current analyses, resulting in a final sample of 40 individuals with SCI.

The AB group comprised 32 participants recruited from the community via convenience sampling, with similar demographics to the SCI group between 2021 and 2022. The exclusion criteria were the same as those used for the SCI group.

### Study measures

Demographic details of both groups were collected at the beginning of the study. These factors included age, sex, and years of education. Injury characteristics included level of injury and completeness of the cord lesion, using the American Spinal Injury Association Impairment Scale (AIS), and conducted by a medical specialist based on the International Standards for Neurological Classification of SCI (http://ais.emsci.org). To evaluate differences in EF between the SCI and AB groups, both groups were administered a standardised measure of cognitive functioning. Due to the Covid-19 lockdown, cognitive assessments were performed either in person or online using the Neuropsychiatry Unit Cognitive Assessment Tool (NUCOG) [19]. The NUCOG is a validated cognitive measure that assesses cognitive functioning across five cognitive domains (attention, visuoconstructional, memory, executive function, and language) in various psychiatric and medical conditions such as SCI [19]. The NUCOG has demonstrated criterion, convergent, and discriminant validity in a number of disorders, as well as having acceptable reliability and specificity/sensitivity [19]. NUCOG domains have been shown to be highly correlated to established standard cognitive tests such as the Wechsler Adult Intelligence Scale [19, 20]. The NUCOG was designed to be administered in approximately 20-30 min, providing a total score of 100 and each domain with a sub-score of 20, whereby a higher score favours better outcome. When online administration of the NUCOG is necessary, reasonable adjustments to the assessment procedures are enacted to satisfy the telehealth environment [18].

### Study procedure

Specifically, we chose to examine and compare NUCOG tasks that require complex EF and minimal EF demands. In total, we focused on six EF tasks: Digit Span Reverse, Calculation, Categorical Fluency, Sequencing, Abstraction, and Interference. For more details see Table 1.

**Table 1:** NUCOG Subtests and Score Interpretation.

Task	Brief Description	Score Interpretation
**EF Tasks**		
Digit Span Reverse	Assesses auditory working memory and mental manipulation of information.	Higher scores (range 0–4) reflect stronger working memory and mental manipulation.
Calculation	Measures numerical reasoning and the ability to manipulate numbers mentally.	Higher scores (range 0–4) indicate stronger numerical reasoning and arithmetic skills.
Categorical Fluency	Assesses verbal productivity and executive control by generating words in a semantic category.	Higher scores (range 0–10) suggest better verbal fluency and executive control.
Sequencing	Evaluates planning and cognitive flexibility by arranging concepts or actions in logical order.	Higher scores (range 0–4) reflect greater ability to plan and organize sequential tasks.
Abstraction	Tests conceptual reasoning and the ability to identify similarities and relationships.	Higher scores (range 0–4) indicate stronger abstract and conceptual reasoning skills.
Interference	Assesses the ability to inhibit automatic responses and manage conflicting information.	Higher scores (range 0–2) suggest better inhibitory control and reduced distractibility.
**m-EF Tasks**		
Digit Span Forward	Assesses immediate auditory attention and basic short-term memory.	Higher scores (range 0–4) indicate better attention span and auditory memory capacity.
Drawing Reproduction	Evaluates visuospatial constructional ability by copying a geometric figure.	Higher scores (range 0–4) indicate more accurate visual-motor integration.
Naming	Assesses confrontational naming and lexical retrieval through object identification.	Higher scores (range 0–5) reflect stronger expressive language and lexical access.
Days of the Week Reverse	Measures overlearned sequencing through reverse recitation.	Higher scores (range 0–7) indicate stronger sequencing ability and cognitive flexibility.

*EF* executive function, *m-EF* minimal executive function.

Calculation was included as an EF measure because mathematical performance relies on executive processes including working memory, planning, inhibition, and cognitive flexibility [21–23]. Similarly, Digit Span Reverse was included as it requires activation of complex processes involving transformation of information that relies upon working memory [24]. Importantly, Digit Span Reverse has distinct differences in the underlying cognitive processes that are involved in Digit Span Forward (DSF), despite being grouped in the same Attention domain of the NUCOG [24, 25].

Four tasks believed to require minimal EF were chosen for comparison to highlight that the cognitive challenges associated with SCI are specific to EF rather than more globally across areas such as language or visuoconstructional domains. These were referred to as minimal EF (m-EF) tasks and included: drawing reproduction, naming, days of the week in reversal, and DSF. Drawing reproduction and naming were chosen as they are tasks from distinct domains that have been shown to have minimal deficits in persons with SCI [5].

Although Days of the Week Reverse and DSF are included within the NUCOG Attention domain, they were classified as relatively lower executive demand tasks. Reverse recitation of days of the week requires some executive processing, including sequencing and working memory; however, it relies largely on retrieval and manipulation of highly overlearned information and places substantially lower executive demands than tasks requiring abstraction, generative fluency, cognitive flexibility, or novel problem-solving. Similarly, DSF involves simple attention and immediate recall without active manipulation of information and is therefore considered a measure of basic attentional capacity rather than higher-order executive functioning [24, 25].

The administration protocols for participants with SCI followed those described by Sandalic et al., 2020 [18].

### Statistical power

Based on a medium multivariate effect size of f²(V) = 0.15, using a Multivariate Analysis of Covariance (MANCOVA), an α probability of 0.05, and a total sample size of 72, post hoc statistical power was estimated using GPower (MANOVA: Global Effects procedure) [26]. Because GPower does not include a dedicated MANCOVA module, the Multivariate Analysis of Variance (MANOVA) global effects option was used as an approximation of the multivariate general linear model [26].

For the six-variable (EF) model, estimated power was 63%, and for the four-variable minimal EF (m-EF) model, estimated power was 72%. A medium multivariate effect size was selected to reflect expected group differences in executive performance within clinical populations, while accounting for variability inherent in cognitive outcomes. This choice balances the need to detect meaningful differences with practical sample-size constraints. Effect sizes in this range are common to ensure sensitivity to clinically relevant differences without overestimating effect magnitude.

### Statistical analysis

MANCOVA was used to examine group differences in cognitive performance while controlling for age, sex, and years of education. Two separate MANCOVA models were conducted to investigate performance across varying EF demands.

The first model included six EF tasks (Digit Span Reverse, Calculation, Sequencing, Categorical Fluency, Abstraction, and Interference) as dependent variables. The second model included four m-EF tasks (Drawing Reproduction, Naming, Days of the Week Reverse, and Digit Span Forward) as dependent variables. Group (SCI vs AB) was entered as the fixed factor, and age, sex, and years of education were included as covariates.

Where significant multivariate effects were identified, follow-up ANCOVAs were conducted. Holm–Bonferroni correction was applied within each task set to control for Type I error.

Independent-samples t-tests were used to examine group differences in continuous demographic variables, and a chi-square test of independence was used to compare sex distribution between groups. Statistical significance was set at *p* < 0.05.

## Results

### Demographic characteristics

The SCI group had a mean (SD) age of 50.0 (18.4) years; 75% were male and the mean (SD) years of education was 12.3 (2.3). The AB group had a mean (SD) age of 51.2 (13.2) years; 62.5% were male and the mean (SD) years of education was 13.1 (2.2). There were no significant group differences in age, *t*(70) = 0.31, *p* = 0.757, years of education, *t*(70) = 1.53, *p* = 0.129, or sex distribution, χ²(1) = 1.28, *p* = 0.258 (see Table 2). The SCI group included predominantly incomplete injuries (80%), with 52.5% cervical and 47.5% thoracic/sacral injuries (see Table 2).

**Table 2:** Descriptive statistics for both groups.

Variable	SCI group (n = 40)	AB group (n = 32)	Statistic	p value
Age, mean (SD)	50.0 (18.4)	51.2 (13.2)	t(70) = 0.31	0.757
Sex (male), n (%)	30 (75)	20 (62.5)	χ²(1) = 1.28	0.258
Years of education, mean (SD)	12.3 (2.3)	13.1 (2.2)	t(70) = 1.53	0.129
Level of Injury				
C1–C4, n (%)	10 (25)	-	-	-
C5–C8, n (%)	11 (27.5)	-	-	-
T1-S5, n (%)	19 (47.5)	-	-	-
Completeness of injury				
Complete, n (%)	8 (20)	-	-	-
Incomplete, n (%)	32 (80)	-	-	-
*AIS B, n (%)*	6 (18.8)	-	-	-
*AIS C, n (%)*	9 (28.1)	-	-	-
*AIS D, n (%)*	17 (53.1)	-	-	-

*SCI* spinal cord injury, *AB* able-bodied.

*No significant group differences were observed (p* > *0.05)*.

### EF model

A MANCOVA was conducted to examine group differences across six EF tasks (Digit Span Reverse, Calculation, Sequencing, Categorical Fluency, Abstraction, and Interference), controlling for age, sex, and years of education. There was a significant multivariate effect of group, Wilks’ Λ = 0.793, *F*(6,62) = 2.70, *p* = 0.022, indicating that participants with SCI differed from AB participants across EF tasks overall. There were no significant multivariate effects of sex (*p* = 0.919) or years of education (*p* = 0.168), and age showed a trend-level association with EF performance (*p* = 0.060).

Follow-up ANCOVAs were conducted with Holm–Bonferroni correction applied within the EF task set to control for Type I error. After correction, the SCI group demonstrated significantly lower performance than the AB group on Categorical Fluency, *F*(1,67) = 8.26, *p* = 0.033, partial η² = 0.110, Abstraction, *F*(1,67) = 7.69, *p* = 0.036, partial η² = 0.103, and Digit Span Reverse, *F*(1,67) = 7.14, *p* = 0.038, partial η² = 0.096. Group differences for Calculation did not remain significant after correction (*p* = 0.102, partial η² = 0.065), and no significant differences were observed for Sequencing or Interference (see Table 3).

**Table 3:** Adjusted EF and m-EF task performance (NUCOG) in SCI and AB groups.

Task	SCI group (n = 40) Mean (SD)	AB group (n = 32) Mean (SD)	F (ANCOVA)	p value	Partial η²
**EF Tasks**					
Digit Span Reverse	2.60 (1.35)	3.29 (0.81)	7.14	0.038*	0.096
Calculation	3.47 (0.88)	3.93 (0.24)	4.68	0.102	0.065
Categorical Fluency	9.29 (1.39)	9.85 (0.61)	8.26	0.033*	0.110
Sequencing	2.97 (1.59)	3.41 (0.97)	1.56	0.216	—
Abstraction	3.51 (0.70)	3.91 (0.25)	7.69	0.036*	0.103
Interference	1.91 (0.39)	2.00 (0.00)	1.08	0.303	—
**m-EF Tasks**					
Digit Span Forward	3.17 (1.08)	3.64 (0.57)	3.70	0.058	—
Drawing Reproduction	3.96 (0.17)	3.98 (0.08)	0.13	0.724	—
Naming	4.97 (0.16)	4.91 (0.53)	1.07	0.304	—
Days of the Week Reverse	6.90 (0.50)	7.00 (0.00)	0.80	0.373	—

*NUCOG* neuropsychiatry unit cognitive assessment tool, *SCI* spinal cord injury, *AB* able-bodied.

Group differences were examined using ANCOVA controlling for age, sex, and years of education. Holm–Bonferroni correction was applied within EF and m-EF task sets.

*Significant after Holm–Bonferroni correction (p* < *0.05)*.

### m-EF model

A second MANCOVA examined group differences across four tasks characterised as minimal EF demand (Drawing Reproduction, Naming, Days of the Week Reverse, and Digit Span Forward), controlling for age, sex, and years of education. There was no significant multivariate effect of group, Wilks’ Λ = 0.919, *F*(4,64) = 1.41, *p* = 0.240. Age was significantly associated with performance across these tasks (*p* = 0.046), whereas sex (*p* = 0.762) and years of education (*p* = 0.664) were not significant. Holm-corrected follow-up ANCOVAs indicated no significant group differences on any m-EF task (see Table 3).

## Discussion

This study examined whether adults in the acute and subacute phases following SCI and AB participants differ in performance across tasks imposing varying levels of EF demand. Using two separate MANCOVA models, we demonstrated a significant multivariate group difference across EF tasks, but no significant multivariate group difference across tasks characterised as minimal EF demand. After Holm–Bonferroni correction, the SCI group showed significantly lower performance than AB participants on Categorical Fluency, Abstraction, and Digit Span Reverse. No significant group differences were observed across minimal EF tasks. Together, these findings indicate that cognitive differences associated with SCI are most evident on tasks requiring higher-order EF processes rather than on tasks requiring simple recall or overlearned responses.

The dissociation between Digit Span Forward and Digit Span Reverse is particularly informative. Although both tasks involve attentional processes, Digit Span Reverse imposes greater working memory demands due to the need for storage and manipulation of information. In contrast, Digit Span Forward primarily reflects simple attention and immediate recall. The absence of group differences on Digit Span Forward, coupled with significantly poorer performance on Digit Span Reverse, suggests that individuals with SCI demonstrate relative preservation of simple attentional capacity but reduced efficiency in working memory manipulation. This distinction highlights the importance of differentiating between maintenance and manipulation processes when assessing EF in clinical populations.

Similarly, significant group differences were observed on Categorical Fluency and Abstraction, both of which require generative processing, conceptual reasoning, and flexible cognitive control. These tasks place greater demands on higher-order EF systems than sequencing or interference tasks, where group differences were not statistically significant after correction. Although Calculation did not remain significant following correction, the direction of the effect was consistent with the broader EF pattern, suggesting that tasks involving strategic planning and manipulation may be more sensitive to EF inefficiencies following SCI.

Importantly, no significant group differences were observed across minimal EF tasks, including Drawing Reproduction, Naming, Days of the Week Reverse, and Digit Span Forward. This pattern strengthens the interpretation that cognitive differences in SCI are more evident on tasks requiring increased executive control. This is consistent with previous research indicating that executive function and attention are among the most affected cognitive domains following SCI, while other domains may be relatively less impacted [5]. However, the present findings should be interpreted in the context of the specific tasks examined, rather than as a comprehensive evaluation of all cognitive domains. The absence of differences in minimal EF tasks, despite adequate statistical power within this model, further supports the specificity of EF-related vulnerability.

Collectively, these findings suggest that when cognitive performance is examined across varying executive demands, participants with SCI demonstrate preserved performance on low-demand tasks but reduced performance as executive complexity increases. This has important implications for neurocognitive assessment. Domain-level scoring approaches that aggregate tasks of differing executive demand (for example, combining forward and backward digit spans within an attention or working memory index) may obscure clinically meaningful differences [25]. Although the NUCOG detected group differences in the present study, its domain-level scoring aggregates tasks of varying executive demand, which may obscure clinically meaningful EF-specific deficits and limit sensitivity to higher-order executive impairments. Accordingly, our results highlight the need to evaluate EF components individually, particularly working memory manipulation and generative fluency processes.

From a clinical perspective, reduced EF capacity may affect planning, problem-solving, behavioural regulation, and the acquisition of compensatory strategies—skills central to rehabilitation and adjustment following SCI. Targeted screening approaches that explicitly assess higher-order EF processes may therefore facilitate earlier identification of cognitive vulnerabilities and inform tailored rehabilitation planning. These findings further underscore the need for a targeted neurocognitive screening tool that explicitly assesses higher-order EF domains, including complex attention, working memory, and processing speed [14, 21, 27, 28]. Such domains are directly relevant to real-world daily activities, including managing medication schedules, coordinating appointments, learning and applying rehabilitation strategies, problem-solving unexpected challenges, maintaining attention during therapy sessions, and organising complex self-care routines.

An additional observation was the greater variability in cognitive performance within the SCI group, suggesting heterogeneity in executive functioning. While some individuals performed within normative ranges, others demonstrated more pronounced difficulties, highlighting the importance of individualized assessment and tailored rehabilitation approaches. Accurate identification of EF-specific inefficiencies in these areas may enhance rehabilitation engagement, support adaptive skill acquisition, and ultimately improve functional outcomes following SCI.

Future research should continue to examine EF-specific mechanisms across the acute, subacute, and chronic stages of SCI and explore how these cognitive differences interact with real-world daily living.

### Study limitations

The main limitation of this study was the relatively small sample size in both groups, resulting in a higher risk of Type II errors and consequent problems in detecting significant differences in all EF tasks. However, SCI is a relatively rare neurological condition, and recruiting adults with SCI can be challenging owing to the numerous vulnerabilities they face, especially during the acute phase. Another limitation is the absence of specific processing-speed measures. As described by Lazzaro et al. [14], processing speed deficits are found within the SCI population; however, these deficits are not directly assessed by the NUCOG. The Wechsler Adult Intelligence Scale has short processing speed subtests that include speed and accuracy trade-off tasks that could provide a more direct measure of this domain in the SCI population [20]. However, adjustment for participants with loss of hand movement should be considered. Additionally, due to the Covid-19 pandemic, the study involved a mix of face-to-face and telehealth-completed NUCOG assessments. Despite accommodating reasonable adjustments to the administration of NUCOG online, it is important to recognize the potential differences in participants’ level of attention between the different modalities of online versus face-to-face assessment. Future studies should aim to reconcile this by assessing EF abilities through a single modality, as attention is a core component of executive functions.

An additional limitation relates to the recruitment and comparability of the control group. The able-bodied (AB) sample was recruited via convenience sampling and was not systematically matched to the SCI group on variables such as socioeconomic status, premorbid intellectual functioning, or broader health characteristics. Furthermore, the AB group was recruited over a slightly later time period (2021–2022) compared to part of the SCI cohort (2019–2022), raising the possibility of unmeasured cohort effects, including those associated with the COVID-19 pandemic. While no significant group differences were observed in age, sex, or years of education, the absence of a premorbid intelligence measure limits the ability to fully account for baseline cognitive differences between groups. These factors should be considered when interpreting group comparisons. Finally, the findings of this study are based on individuals assessed during the acute and subacute phase of SCI and may not generalise to those in later, chronic stages of injury, where patterns of cognitive functioning may differ over time.

Future research should consider incorporating a longitudinal comparison of EF domains between the SCI and AB groups. This could help to identify whether these deficits are consistent across different stages of recovery. If EF does not recover over time, this may suggest that SCI is related to accelerated cognitive aging and this needs to be accounted for in rehabilitation strategies.

## Conclusions

These findings provide important insight into the EF challenges experienced by adults with SCI. When examined across varying levels of cognitive demand, individuals in the acute and subacute phases following SCI demonstrate preserved performance on low-demand tasks but reduced performance as executive complexity increases. This pattern highlights the importance of identifying EF-specific inefficiencies, particularly in working memory manipulation, generative fluency, and higher-order cognitive control.

Early identification of these deficits is critical, as executive functions underpin key processes required for successful rehabilitation, including planning, problem-solving, behavioural regulation, and the acquisition of compensatory strategies. While brief cognitive screening tools such as the NUCOG can detect group-level differences, their domain-level scoring may obscure clinically meaningful EF-specific impairments.

Accordingly, there is a need for targeted neurocognitive screening approaches that explicitly assess higher-order executive processes. Improving the precision of EF assessment in this population may enhance rehabilitation engagement, support adaptive functioning, and ultimately improve long-term outcomes for individuals living with SCI [2, 21, 27–30].

### Transparency, rigor and reproducibility summary

Data were collected between 2019 and 2022. The sample sizes reflect the number of individual participants who completed the cognitive assessments (n = 40 SCI, n = 32 AB controls). The effect sizes of the primary and secondary outcomes are reported in the main text. Ethical approval was obtained from the relevant committees, and the study was prospectively registered with the Australian and New Zealand Clinical Trials Registry (ACTRN12619001702101). All participants provided informed consent before participation. De-identified data from this study are not currently available in a public archive, but may be provided by emailing the corresponding author, subject to ethical approval and data-sharing agreements. The analytic code used in the study is not stored in a public repository but may be shared upon request. The full article is available upon request from the corresponding author.

## Data Availability

The datasets generated are available from the corresponding author upon reasonable request.
